# Characterizing the electrical properties of raised S/D junctionless thin-film transistors with a dual-gate structure

**DOI:** 10.1186/1556-276X-9-669

**Published:** 2014-12-11

**Authors:** Ya-Chi Cheng, Hung-Bin Chen, Jun-Ji Su, Chi-Shen Shao, Cheng-Ping Wang, Chun-Yen Chang, Yung-Chun Wu

**Affiliations:** Department of Engineering and System Science, National Tsing Hua University, 101, Section 2, Kuang Fu Road, Hsinchu, 30013 Taiwan; Department of Electronics Engineering, Institute of Electronics, National Chiao Tung University, 1001, Ta Hsueh Road, Hsinchu, 30013 Taiwan

**Keywords:** Junctionless (JL), Thin-film transistor (TFT), Raised source-and-drain (raised S/D), Dual-gate, Reliability

## Abstract

This letter demonstrates a p-type raised source-and-drain (raised S/D) junctionless thin-film transistors (JL-TFTs) with a dual-gate structure. The raised S/D structure provides a high saturation current (>1 μA/μm). The subthreshold swing (SS) is 100 mV/decade and the drain-induced barrier lowering (DIBL) is 0.8 mV/V, and the *I*_on_/*I*_off_ current ratio is over 10^8^ A/A for *L*_g_ = 1 μm. Using a thin channel structure obtains excellent performance in the raised S/D structure. Besides the basic electrical characteristics, the dual-gate structure can also be used to adjust *V*_th_ in multi-*V*_th_ circuit designs. This study examines the feasibility of using JL-TFTs in future three-dimensional (3D) layer-to-layer stacked high-density device applications.

## Background

Recently, the concept of the junctionless (JL) field-effect transistor (FET), which contains a heavily, uniformly, and homogeneously doping species in the channel and source/drain (S/D), has been intensively studied [1–4]. The JL device is intrinsically a gated resistor, i.e., a resistor with a gate for controlling the carrier density and the current flow. The advantages of JL devices include (1) avoidance of the use of an ultra-shallow source/drain junction, which greatly simplifies the process flow, (2) low thermal budgets owing to implant activation annealing after the gate stack formation is eliminated, and (3) the current transport is in the bulk of the semiconductor, which reduces the impact of imperfect semiconductor/insulator interfaces. These features have also been demonstrated with poly-Si thin-film transistor (TFT) [5–7], which are suitable for monolithic three-dimensional (3D) vertically stacked integrated circuits (ICs), which continue the applicability of Moore’s law [8]. However, the JL channel thickness should be thin enough to turn off the JL devices. This limits the saturation current of the junctionless thin-film transistor (JL-TFT) [7, 9]. Meanwhile, it adversely increases series resistance in the S/D and decreases drain current. In order to conquer this issue, the raised source-and-drain (raised S/D) structure is used for this works.

In this work, the thin-channel structure trimmed by oxidation and HF is used for turning off the devices, and the raised S/D structure is built for high saturation current. A dual-gate structure can be applied in multi-threshold voltage (multi-*V*_th_) applications [10], and its temperature is discussed for the p-type raised S/D JL-TFTs.

## Methods

### Device fabrication and experiment

**Figure 1:**
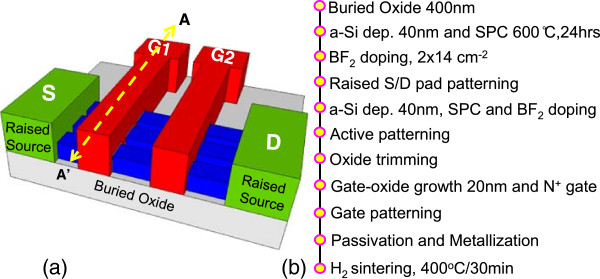
**Device structure and detailed process flow of the fabrication. (a)** The device structure for the raised S/D p-type JL-TFTs. **(b)** The detailed process flow of the fabrication in the raised S/D JL-TFTs. The positions A and A’ indicate the cross-section of the channel.

Figure 1a schematically presents the proposed device structure of the raised S/D JL-TFT, and Figure 1b shows the detailed process flows of the fabrication in the raised S/D JL-TFT. The p-type raised S/D JL-TFT is fabricated by initially growing a 400-nm thermal silicon dioxide layer on a 6-in. silicon wafer. A 40-nm amorphous Si (a-Si) layer was deposited by low-pressure chemical vapor deposition (LPCVD) at 550°C. Then, the a-Si layer was formed by solid-phase recrystallized (SPC) process at 600°C for 24 h in nitrogen ambient. After borondifluoride (BF_2_) ion implantation with 30 keV at a dose of 2 × 10^14^ cm^−2^ for the p^+^ raised S/D doping followed by furnace annealing at 600°C for 4 h, the raised S/D is patterned by e-beam lithography. Subsequently, using the same method for the production of a-Si deposition (40 nm), the implantation (30 keV, BF_2_, 2 × 10^14^ cm^−2^) and the SPC process form the channel layer. While serving as a channel, the active layer was patterned by e-beam lithography and then anisotropically etched by time-controlled reactive ion etching (RIE). The patterned width of each nanosheet channel is 0.3 μm. Then, the active channel was mesa-etched by time-controlled wet etching of dilute HF to form the omega-shaped channel. Next, a sacrificial oxide as a trimming process was thermally grown at 900°C for 2 h, which consumes around 22-nm-thick poly-Si. Subsequently, the dry oxide of 20-nm thickness was deposited as the gate oxide layer, consuming around 10-nm-thick poly-Si to form a 7.35-nm-thick channel. The 150-nm-thick *in situ* doped n^+^ poly-silicon was deposited as a gate electrode and patterned by e-beam and RIE. Additionally, a 200-nm SiO_2_ passivation layer was deposited. Finally, a 300-nm-thick Al-Si-Cu metallization was performed and sintered at 400°C for 30 min.

## Results and discussion

**Figure 2:**
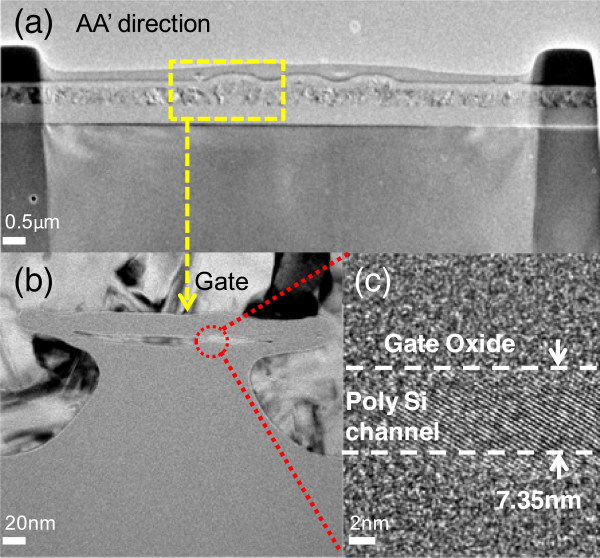
**TEM images of the raised S/D channel. (a)** The cross-sectional TEM images of the raised S/D channel along the AA’ direction with *L*_g_ = 0.5 μm. **(b)** The enlarged TEM images in **(a)** with omega-gate structure. **(c)** The enlarged TEM images in **(b)** with *T*_ch_ = 7.35 nm and fin width = 0.3 μm.

**Figure 3:**
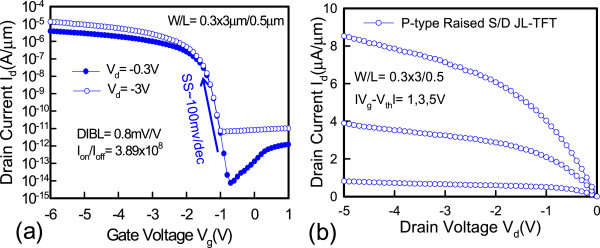
***I***_**d**_ **-** ***V***_**g**_ **characteristics and** ***I***_**d**_ **-** ***V***_**d**_ **curve. (a)** The transfer *I*_d_-*V*_g_ characteristics and **(b)** *I*_d_-*V*_d_ curve in the raised S/D JL-TFTs with *L*_g_ = 0.5 μm at *V*_d_ = −0.3 V.

**Figure 4:**
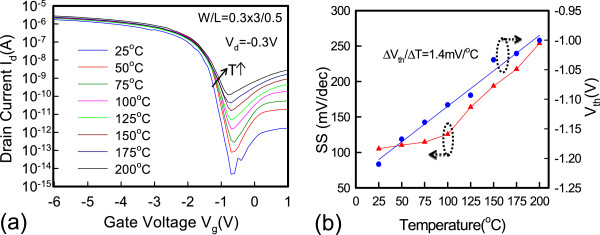
**Temperature dependence and measured SS and threshold voltage. (a)** Temperature dependence (25°C to 200°C) on *I*_d_-*V*_g_ characteristics at *V*_d_ = −0.3 V for the raised S/D JL-TFTs. **(b)** The dependence of sub-threshold swing (SS) and threshold voltage (*V*_th_) between various temperatures for the p-type raised S/D devices.

Figure 2a shows the cross-sectional transmission electron microscopic (TEM) image along the AA’ direction, as shown in Figure 1a. Figure 2b,c displays the enlarged images of Figure 2a. The nano-sheet channel of the raised S/D structure is covered by the omega-gate electrode, which is expected to improve electrostatic gate control and achieve superior performance [11]. Figure 2c clearly shows that the nano-sheet channel thickness is 7.35 nm, and the TEM photograph shows that the single-crystal-like channel with large grain size is expected to achieve superior performance because of the oxidation trimming process. The channel region is doped with a boron concentration of 4 × 10^19^ cm^−3^. Figure 3a plots the transfer *I*_d_-*V*_g_ characteristics of the raised S/D JL-TFT with *L*_g_ = 0.5 μm. The on current (*I*_on_) is defined as the drain current at *V*_g_ = −6 V for the raised S/D JL-TFT. The off current (*I*_off_) is defined as the lowest drain current. The excellent transfer characteristics of the raised S/D JL-TFT with *L*_g_ = 0.5 μm include the following: (1) subthreshold swing (SS) = 100 mV/decade, (2) DIBL = 0.8 mV/V, and (3) *I*_on_/*I*_off_ current ratio = 3.85 × 10^8^. Figure 3b shows the *I*_d_-*V*_d_ output characteristics of the raised S/D JL-TFT. The on resistance is low at various over-drive voltages with the raised S/D structure. Figure 4a demonstrates the temperature dependence of the raised S/D JL-TFT. Based on the *I*_d_-*V*_g_ curves in Figure 4a, Figure 4b presents the measured SS and threshold voltage (*V*_th_) as functions of temperature at *V*_d_ = −0.3 V. This figure reveals that, as the temperature increases, the absolute *V*_th_ value decreases and the SS increases. The positive shifting of *V*_th_ for the raised S/D device is discussed in Figure 4b. The *V*_th_ is defined as the gate voltage at *I*_d_ = 10^−9^ A. The *V*_th_ equation could be presented as the following [12]:
1

where *V*_fb_ is the flat-band voltage, *C*_ox_ is the gate capacitance per unit of length, *N*_A_ is the carrier concentration, *W*_D_ is the depletion width, *W*_ch_ is the effective channel width, *T*_ch_ is the channel thickness, and *m*_h_^*^ is the effective mass of the confined holes. When the device is heated, the bandgap (*E*_g_) decreases. Therefore, *N*_A_ increases. For the above reasons, absolute *V*_th_ is expected to decrease with increasing temperature. Notably, the temperature dependence of the threshold voltage for the poly-channel device is 1.4 mV/°C. This value is close to the *V*_th_ temperature dependence of the single-crystal channel (1.65 mV/°C) [13].

**Figure 5:**
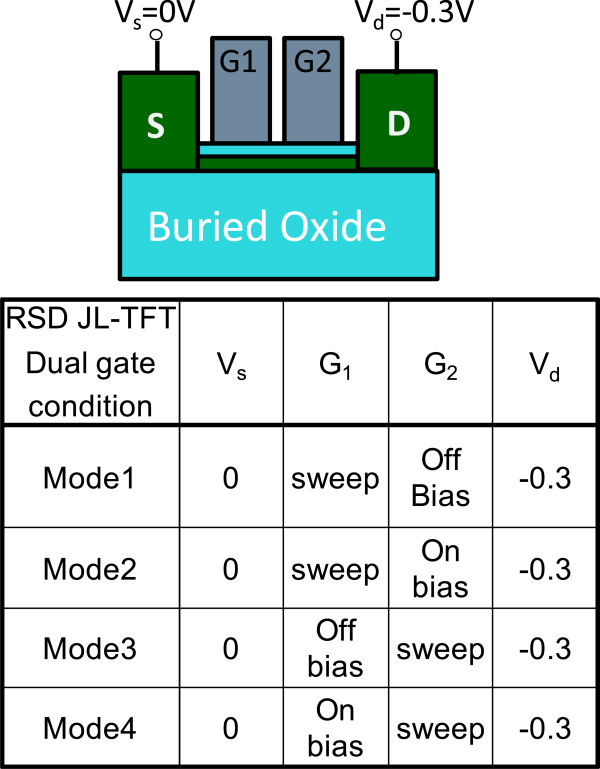
**Schematic of the measured raised S/D devices with dual-gate structure.** The various modes at different bias conditions for the dual-gate structure.

**Figure 6:**
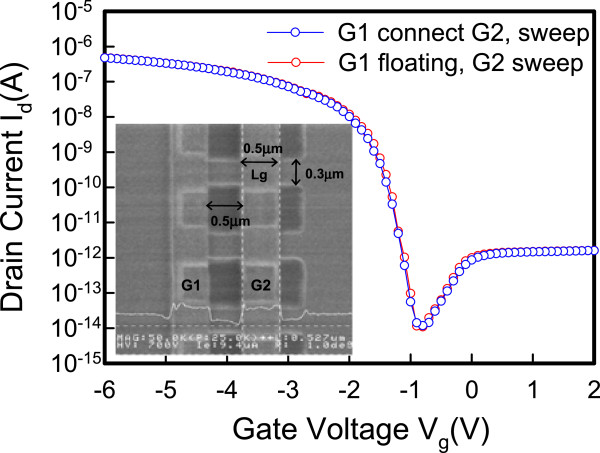
**The transfer** ***I***_**d**_ **-** ***V***_**g**_ **characteristics of different gate connections.** The inset shows the SEM image of the dual-gate structure.

**Figure 7:**
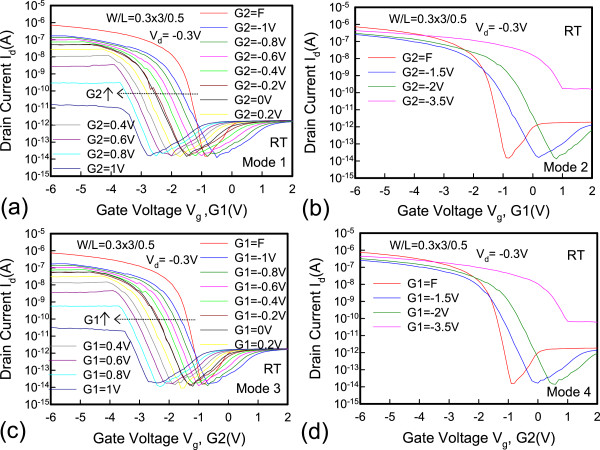
**The transfer** ***I***_**d**_ **-** ***V***_**g**_ **characteristics for various modes (a-d), listed in Figure** 5 **.**

**Figure 8:**
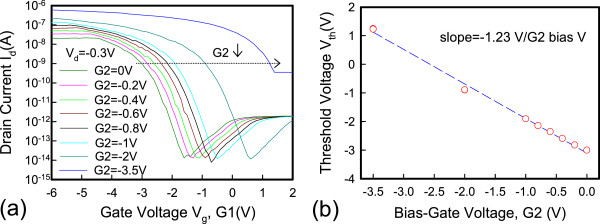
**Transfer** ***I***_**d**_ **-** ***V***_**g**_ **characteristics and G2 bias dependence. (a)** The transfer *I*_d_-*V*_g_ characteristics at different G2 gate bias. **(b)** The impact of G2 bias dependence on the *V*_th_ for the raised S/D JL-TFTs.

**Figure 9:**
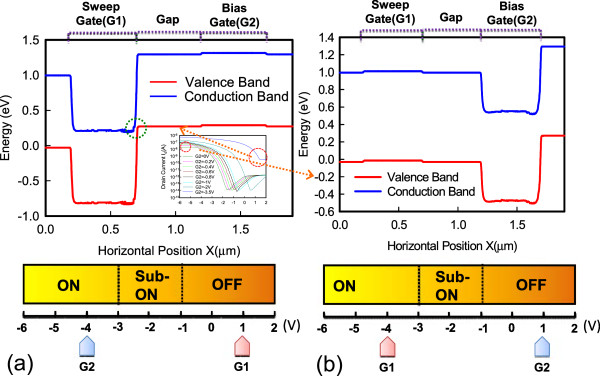
**Simulation results at (a) G1 = 1 V, G2 = −4 V for mode 2 situation and (b) G1 = −4 V, G2 = 1 V for mode 1 situation.**

**Figure 10:**
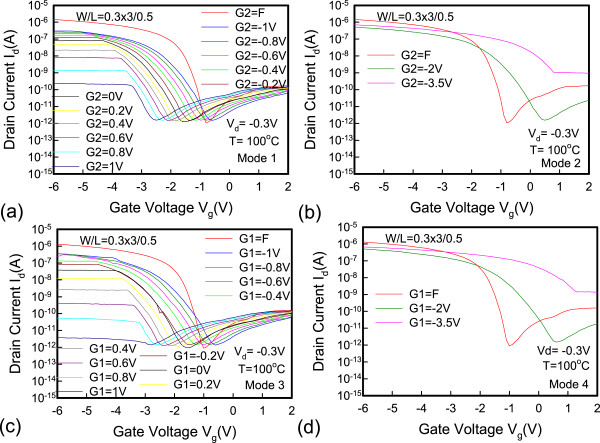
**The temperature dependence of dual-gate JL-TFTs at 100°C on** ***I***_**d**_ **-** ***V***_**g**_ **characteristics (a-d).**

Figure 5 shows the dual-gate structure with different operation modes. In mode 1, G1 is sweeping, G2 has an off-state bias condition, and *V*_d_ is applied at −0.3 V. In mode 2, G1 is sweeping, and G2 has an on-state bias condition. The reverse conditions for G1 and G2 occur in mode 3 and mode 4. Figure 6 shows the *I*_d_-*V*_g_ characteristics. The red line represents that G1 is floating and G2 is sweeping. The blue line represents that G1 connects G2 and they are sweeping simultaneously. The experimental data show a good match in the *I*_d_-*V*_g_ curves, which indicates that the series resistance between G1 and G2 is insignificant and does not degrade electrical performance. The inset in Figure 6 shows the scanning electron microscope (SEM) image of the dual-gate structure. The distance between the dual gate is 0.5 μm. Figure 7 depicts the *I*_d_-*V*_g_ curves for different operation modes. The electrical performances for mode 1 and mode 2 are similar to those for mode 3 and mode 4 at *V*_d_ = −0.3 V. Figure 7a,c shows that, when the G2 and G1 approaches off-state bias condition, the on current is clearly pinning and absolute *V*_th_ is increasing. Figure 7b,d shows that, when the G2 and G1 approaches on-state bias condition, the on current is increasing and the absoute *V*_th_ is decreasing. In Figure 8a, the *V*_th_ can be adjusted by the dual-gate structure applying different gate bias. In Figure 8b, the *V*_th_ sensitivity of G2 bias is approximately 1.23 V/V, and the experimental data show that the relationship is linear. The detailed results are discussed by 3D TCAD simulation in Figure 9. To obtain accurate numerical results for a nanometer-scale device, the device is simulated by solving 3D quantum-corrected equations using the commercial tool, Synopsys Sentaurus Device [14]. In quantum-corrected equations, a density gradient model is used in the simulation, as listed below [15, 16]:
2

where *n* is the electron concentration, Nc is the effective density of states of the conduction band (Ec), *F*_1/2_ is the Fermi-Dirac integral, μn is the effective mass of the electron, and *T*_*n*_ is the electron temperature. The bandgap narrowing model, the band-to-band tunneling model, and the Shockley-Read-Hall recombination with the doping-dependent model are also considered. The direct tunneling model is not utilized because high-*k*/metal-gate technology is used. The mobility model used in the device simulation is according to Mathiessen’s rule, which is expressed as
3

where *D* = exp(*x/l*_crit_), *x* is the distance from the interface, and *l*_crit_ is a fitting parameter. The mobility consists of three parts: 1) surface acoustic phonon scattering (*μ*_surf_aps_), 2) surface roughness scattering (*μ*_surf_rs_), and 3) bulk mobility with doping-dependent modification (*μ*_bulk_dop_). The details are described in [14, 17]. Figure 9a shows the high off current when G1 is sweeping at 2 V and G2 is at on-state bias of −3.5 V. When the G2 is in extreme on-state, band-to-band tunneling occurs easily according to the simulation results. Figure 9b shows the pinning mechanism when G1 is sweeping and G2 is at off-state bias. When the G2 voltage approaches off-state, the valence band will be dropped off, which retards the hole transport and causes a saturation current. Figure 10 shows the temperature characteristics of the dual-gate structure. High-temperature performance is similar to that at room temperature.

## Conclusions

This work realizes the p-type raised S/D JL-TFTs and dual-gate structure. In our devices, the thin channel formed by the oxidation trimming process and raise S/D structure are used. Due to these two ideas, the high on current (>1 μA/μm), low off current (10^−14^ A), and small SS (100 mV/decade) could be achieved. It is a promising structure to get a good-performance JL device and conquer the low-*I*_on_ issue of JL devices. The temperature of the raised S/D devices is discussed for the electrical parameters (SS, *V*_th_). It is worthy to notice that the dual-gate structure can be used to adjust *V*_th_ to fulfill the multi-*V*_th_ circuit designs. The devices are highly promising for future further scaling and 3D stacked IC applications.
